# Synthesis and crystal structure of a new chiral α-amino­oxime nickel(II) complex

**DOI:** 10.1107/S2056989021010537

**Published:** 2021-10-19

**Authors:** Yasmina Homrani, Abdelaziz Dahdouh, Mohamed Amin El Amrani, Pauline Loxq, Frédéric Capet, Isabelle Suisse, Mathieu Sauthier

**Affiliations:** a Laboratoire de Chimie Organique Appliquée, Faculté des Sciences, BP 2121, Université Abdelmalek Essaadi, Tétouan, Morocco; b Univ. Lille, CNRS, Centrale Lille, Univ. Artois, UMR 8181, UCCS, Unité de Catalyse et Chimie du Solide, F-59000, Lille, France

**Keywords:** Nickel, α-amino­oxime, (*R*)-limonene, crystal structure

## Abstract

A dinuclear nickel complex with (*S*)-limonene based amino­oxime ligand has been isolated and its crystal structure determined. The resolved structure of dichloridobis­{(2*S*,5*R*)-2-methyl-5-(prop-1-en-2-yl)-2-[(pyridin-2-yl)methyl­amino]­cyclo­hexan-1-one oxime}dinickel(II), [Ni_2_Cl_2_(C_16_H_23_ClN_3_O)_2_], at 100 K has monoclinic (*P*2_1_) symmetry. The two Ni^II^ ions in the dinuclear complex are each coordinated in a distorted octa­hedral environment by three nitro­gen atoms, a terminal chloride and two μ bridging chlorides. Each oxime ligand is coordinated to nickel(II) by the three nitro­gen atoms, leading to two five-membered chelate rings, each displaying an envelope conformation. In the crystal, numerous inter­molecular and intra­molecular hydrogen bonds lead to the formation of a three-dimensional network structure.

## Abstract 2

The reaction of a nickel precursor with an enanti­omerically pure amino-oxime issued from (*R*)-limonene led to the formation of bis­[κ^3^ *N*,*N*,*N*-(amino­oxime)-μ-chlorido]­dichloro­dinickel as a new dinuclear nickel complex.

## Chemical context

Asymmetric synthesis allows the preparation of enanti­omerically enriched compounds either by using a chiral auxiliary, which will be temporarily introduced, or by using catalytic procedures (Gawley & Aubé, 2012 ▸). This latter method is particularly attractive as it contributes to the development of green chemistry, which maximizes efficiency and minimizes haza­rdous effects on human health and the environment (Anastas & Zimmerman, 2013 ▸). Thus, asymmetric catalysis avoids synthetic steps and only catalytic amounts of the optically pure auxiliary are needed (Ojima, 2010 ▸). As part of the development of this chemistry, the synthesis of new chiral organometallic complexes is always challenging. The pivotal point is then the synthesis of optically pure ligands, which will be coordinated to the metal center. In terms of sustainable chemistry, using the chiral pool to develop new ligands is most inter­esting (Elalami *et al.*, 2015 ▸). Coord­ination metal complexes containing terpenoid fragments are widely used in the pharmaceutical field and in catalysis. We have therefore developed ligands based on terpenes such as pinene and limonene (El Alami *et al.*, 2009 ▸, 2015 ▸; Chahboun *et al.*, 2012 ▸). In particular, the synthesis of optically pure amino-oxime ligands has been performed successfully from (*R*)-limonene (El Alami *et al.*, 2012 ▸). These compounds possess structures with two or three nitro­gen atoms as donor heteroatoms that could coordinate to the metal center. They have advantageously replaced phosphine ligands, which are generally unstable under air. Ruthenium (Benabdelouahab *et al.*, 2015 ▸) and palladium (de la Cueva-Alique *et al.*, 2019 ▸) complexes have already been synthezised with these ligands. Here we report the first synthesis of a limonene-based α-amino­oxime nickel complex and its crystal structure. In the dinuclear title complex, each nickel ion is coordinated by (1*S*,4*R*)-1-picolyl­amino-*p*-menth-8-en-2-one oxime. The ligand was first synthesized from (*R*)-limonene through the addition of nitrosyl chloride, NOCl, to a picolyl­amine moiety, allowing the formation of the oxime moiety. 
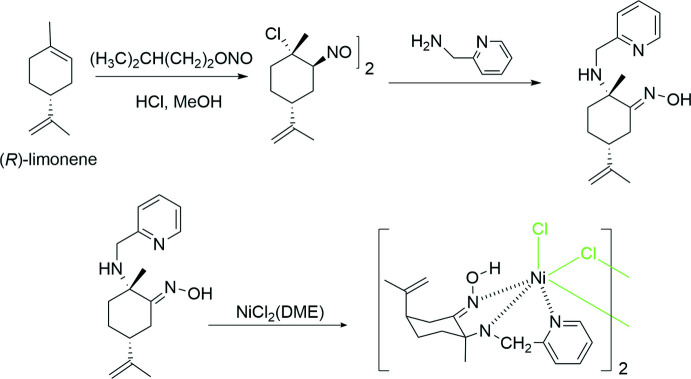


## Structural commentary

**Figure 1:**
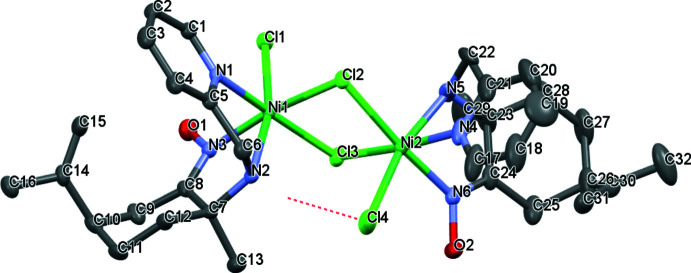
Displacement ellipsoid plot at the 50% probability level for Ni_2_(amino-oxime)_2_Cl_4_. H atoms are omitted for clarity.

The title compound (Fig. 1 ▸) crystallizes in the monoclinic space group *P*2_1_ with two chiral mol­ecules per unit cell. The two Ni^II^ ions in the dinuclear complex are each coordinated by three nitro­gen atoms, a terminal chloride and two μ bridging chlorides. The environment around each metal center can then be described as a distorted octa­hedron with N1—Ni1—N2 and Cl1—Ni1—Cl3 angles of 79.91 (13) and 91.99 (4)°, respectively, together with Cl1—Ni1—N2 and Cl2—Ni1—N1 angles of 165.04 (11) and 88.69 (10)°, respectively. A similar arrange­ment can be found around the Ni2 atom [N4—Ni2—N5, Cl2—Ni2—Cl4, Cl4—Ni2—N5 and Cl4—Ni2—N4 = 79.7 (2), 99.38 (4), 166.04 (12) and 93.24 (16)°, respectively].

Each amino­oxime ligand is coordinated to nickel(II) by the three nitro­gen atoms, leading to two five-membered chelate rings, each displaying an envelope conformation (with N2 as the flap for Ni1/N1/C5/C6/N2 and N5 for Ni2/N4/C21/C22/N5). The six-membered carbocycles of the limonene units adopt a chair conformation. The lengths of the Ni1—N1, Ni1—N2 and Ni1—N3 bonds are 2.077 (3), 2.126 (4) and 2.041 (3) Å, respectively, while Ni2—N4, Ni2—N5 and Ni2—N6 are 2.095 (4), 2.103 (4) and 2.027 (3) Å. Atoms Cl1 and Cl4 are in a *trans*-position at distances of 2.4408 (12) and 2.4077 (14) Å from the metal centers Ni1 and Ni2, respectively. The two metal centers are linked by two bridging Cl atoms with an average Ni—Cl distance of 2.42 Å, which is normal for these bond lengths. All these values compare well with literature values. The two nickel ions are separated by a distance of 3.5198 (7) Å, which is similar to average values (Zheng *et al.*, 2010 ▸; Cheng *et al.*, 2012 ▸).

## Supra­molecular features

**Figure 2:**
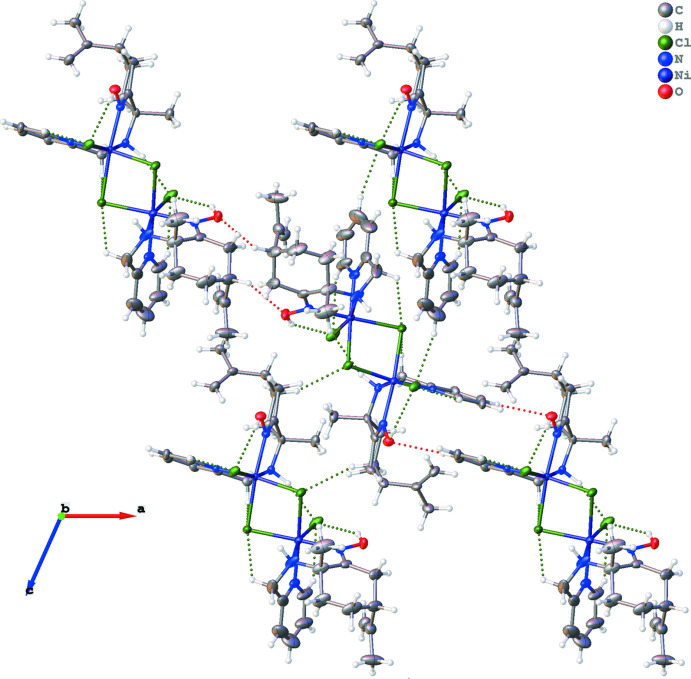
Inter­molecular and intra­molecular hydrogen bonds in the structure, shown as dashed lines.

The crystal structure is stabilized by numerous inter­molecular and intra­molecular hydrogen bonds (Table 1 ▸), which link the component into a three-dimensional network (Figs. 2 ▸ and 3 ▸). In particular, the two {Ni(aminoxime)μ-Cl}Cl units are slightly asymmetrical with the existence of a hydrogen-bonding inter­action between the amine N2—H2 linked to Ni1 and the chlorine atom Cl4 linked to Ni2. In addition, the two oxygen atoms O1 and O2 of the oxime groups are involved in intra­molecular O1—H1⋯Cl1 and O2—H2*A*⋯Cl4 hydrogen bonds and in inter­molecular C3—H3⋯O1 and C26—H26⋯O2 inter­actions.

## Database survey

The amino­oxime ligand used in this study was previously reacted with palladium and platinum precursors, generating three *N*-coordinated cationic complexes as enanti­opure compounds (de la Cueva-Alique *et al.*, 2019 ▸). A heteronuclear Ti^IV^/Pd^II^ complex has also been described. The compounds were studied to assess their potential biological activity, a high anti­cancer activity (de la Cueva-Alique *et al.*, 2019 ▸).

## Synthesis and crystallization

To a solution of Ni^II^ chloride ethyl­ene glycol dimethyl ether (0.15 g, 1.48 mmol) in MeOH (5 mL) was added (1*S*,4*R*)-1-picolyl­amino-*p*-menth-8-en-2-one-oxime (0.101 g, 0.36 mmol) dissolved in MeOH (3 mL). The solution turned green. The mixture was stirred overnight at room temperature during which time the mixture changed color to blue–green. The solvent was then evaporated to produce a crude solid that was washed with diethyl ether before crystallization. Single crystals were grown by slow diffusion at room temperature of diethyl ether into a di­chloro­methane solution. Elemental analysis calculated for C_32_H_46_Cl_4_N_6_Ni_2_O_2_: C, 46.33; H, 5.54; N, 9.65. Found: C, 46.35; H, 5.672; N, 9.77.

## Refinement

Crystal data, data collection and structure refinement details are summarized in Table 2 ▸. N- and O-bound atoms were refined with the restraint *U*_iso_(H) = 1.2*U*_eq_(N) or 1.5*U*_eq_(O). H atoms were positioned geometrically(C—H = 0.95–1.00 Å) and refined as riding with *U*_iso_(H) = 1.2*U*_eq_(C) or 1.5*U*_eq_(C-meth­yl)

## Supplementary Material

Crystal structure: contains datablock(s) I. DOI: 10.1107/S2056989021010537/ex2048sup1.cif

Structure factors: contains datablock(s) I. DOI: 10.1107/S2056989021010537/ex2048Isup2.hkl

CCDC reference: 2115017

Additional supporting information:  crystallographic information; 3D view; checkCIF report

## Figures and Tables

**Figure 3:**
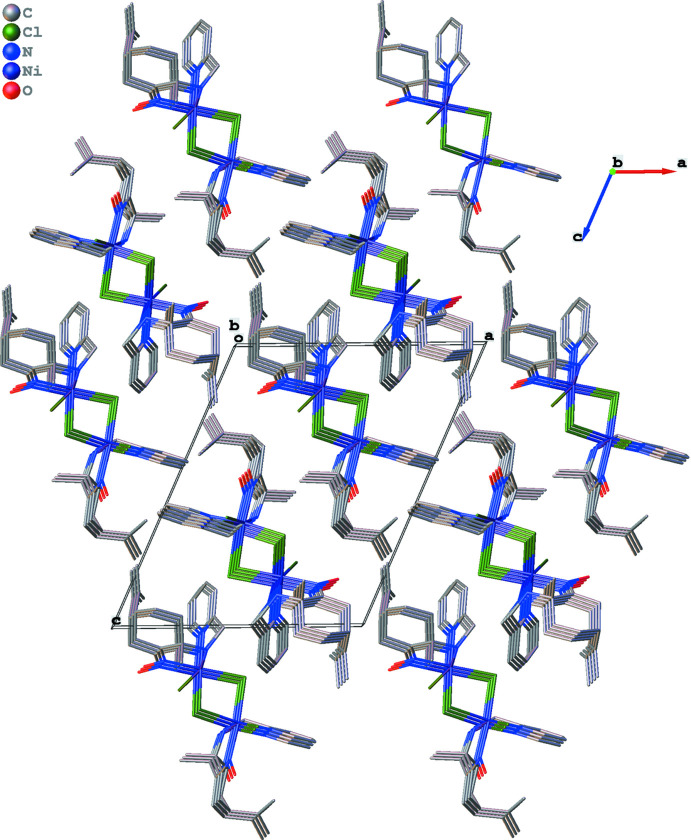
Packing diagram.

**Table 1:** Hydrogen-bond geometry (Å, °)

*D*—H⋯*A*	*D*—H	H⋯*A*	*D*⋯*A*	*D*—H⋯*A*
O1—H1⋯Cl1	0.85 (7)	2.32 (6)	3.009 (4)	139 (6)
N2—H2⋯Cl4	0.77 (5)	2.46 (5)	3.209 (4)	166 (5)
O2—H2*A*⋯Cl4	0.76 (8)	2.31 (7)	2.978 (4)	147 (7)
C3—H3⋯O1^i^	0.95	2.58	3.432 (5)	149
C1—H1*A*⋯Cl1	0.95	2.75	3.369 (5)	124
C6—H6*A*⋯Cl2	0.99	2.76	3.309 (5)	115
C11—H11*B*⋯Cl3^ii^	0.99	2.64	3.573 (5)	156
C17—H17⋯Cl4	0.95	2.69	3.327 (6)	125
C26—H26⋯O2^iii^	1.00	2.56	3.489 (6)	154
C22—H22*B*⋯Cl2	0.99	2.81	3.352 (6)	115
C19—H19⋯Cl1^iv^	0.95	2.64	3.570 (7)	167

Symmetry codes: (i) -x+2, y-{\script{1\over 2}}, -z+1; (ii) -x+1, y-{\script{1\over 2}}, -z+1; (iii) -x, y+{\script{1\over 2}}, -z; (iv) -x+1, y-{\script{1\over 2}}, -z.

**Table 2:** Experimental details

Crystal data	
Chemical formula	[Ni_2_Cl_2_(C_16_H_23_ClN_3_O)_2_]
*M*_r_	805.97
Crystal system, space group	Monoclinic, *P*2_1_
Temperature (K)	100
*a*, *b*, *c* (Å)	13.3729 (9), 8.9363 (7), 16.4248 (16)
β (°)	114.014 (2)
*V* (Å^3^)	1792.9 (3)
*Z*	2
Radiation type	Mo *K*α
μ (mm^−1^)	1.39
Crystal size (mm)	0.21 × 0.17 × 0.12

Data collection	
Diffractometer	Bruker APEXII CCD
Absorption correction	Multi-scan (*SADABS*; Krause *et al.*, 2015 ▸)
*T*_min_, *T*_max_	0.669, 0.746
No. of measured, independent and observed [*I* > 2σ(*I*)] reflections	42747, 10769, 9436
*R*_int_	0.037
(sin θ/λ)_max_ (Å^−1^)	0.714

Refinement	
*R*[*F* ^2^ > 2σ(*F* ^2^)], *wR*(*F* ^2^), *S*	0.043, 0.109, 1.05
No. of reflections	10769
No. of parameters	431
No. of restraints	13
H-atom treatment	H atoms treated by a mixture of independent and constrained refinement
Δρ_max_, Δρ_min_ (e Å^−3^)	1.50, −1.19
Absolute structure	Flack *x* determined using 3850 quotients [(*I* ^+^)−(*I* ^−^)]/[(*I* ^+^)+(*I* ^−^)] (Parsons *et al.*, 2013 ▸)
Absolute structure parameter	−0.009 (4)

Computer programs: *APEX2* and *SAINT* (Bruker, 2019 ▸), *SHELXT* (Sheldrick, 2015*a* ▸), *SHELXL* (Sheldrick, 2015*b* ▸) and *OLEX2* (Dolomanov *et al.*, 2009 ▸).

## supplementary crystallographic information

### Crystal data

**Table:**

[Ni_2_Cl_2_(C_16_H_23_ClN_3_O)_2_]	*F*(000) = 840
*M**_r_* = 805.97	*D*_x_ = 1.493 Mg m^−^^3^
Monoclinic, *P*2_1_	Mo *K*α radiation, λ = 0.71073 Å
*a* = 13.3729 (9) Å	Cell parameters from 9996 reflections
*b* = 8.9363 (7) Å	θ = 2.7–30.0°
*c* = 16.4248 (16) Å	µ = 1.39 mm^−^^1^
β = 114.014 (2)°	*T* = 100 K
*V* = 1792.9 (3) Å^3^	Block, green
*Z* = 2	0.21 × 0.17 × 0.12 mm

### Data collection

**Table:**

Bruker APEXII CCD diffractometer	9436 reflections with *I* > 2σ(*I*)
Radiation source: microfocus sealed X-ray tube	*R*_int_ = 0.037
φ and ω scans	θ_max_ = 30.5°, θ_min_ = 1.4°
Absorption correction: multi-scan (SADABS; Krause *et al.*, 2015)	*h* = −17→19
*T*_min_ = 0.669, *T*_max_ = 0.746	*k* = −12→12
42747 measured reflections	*l* = −23→21
10769 independent reflections	

### Refinement

**Table:**

Refinement on *F*^2^	Secondary atom site location: difference Fourier map
Least-squares matrix: full	Hydrogen site location: mixed
*R*[*F*^2^ > 2σ(*F*^2^)] = 0.043	H atoms treated by a mixture of independent and constrained refinement
*wR*(*F*^2^) = 0.109	*w* = 1/[σ^2^(*F*_o_^2^) + (0.0581*P*)^2^ + 0.9636*P*] where *P* = (*F*_o_^2^ + 2*F*_c_^2^)/3
*S* = 1.05	(Δ/σ)_max_ = 0.001
10769 reflections	Δρ_max_ = 1.50 e Å^−^^3^
431 parameters	Δρ_min_ = −1.18 e Å^−^^3^
13 restraints	Absolute structure: Flack *x* determined using 3850 quotients [(*I*^+^)-(*I*^-^)]/[(*I*^+^)+(*I*^-^)] (Parsons *et al.*, 2013)
Primary atom site location: dual	Absolute structure parameter: −0.009 (4)

### Special details

**Table:**

Geometry. All esds (except the esd in the dihedral angle between two l.s. planes) are estimated using the full covariance matrix. The cell esds are taken into account individually in the estimation of esds in distances, angles and torsion angles; correlations between esds in cell parameters are only used when they are defined by crystal symmetry. An approximate (isotropic) treatment of cell esds is used for estimating esds involving l.s. planes.

### Fractional atomic coordinates and isotropic or equivalent isotropic displacement parameters (Å^2^)

**Table:**

	*x*	*y*	*z*	*U*_iso_*/*U*_eq_	
Ni1	0.66455 (4)	0.50226 (6)	0.35327 (3)	0.01526 (11)	
Ni2	0.41917 (4)	0.48960 (6)	0.15985 (3)	0.01813 (12)	
Cl2	0.61155 (8)	0.45928 (14)	0.19208 (7)	0.0259 (2)	
Cl3	0.48342 (7)	0.60270 (12)	0.30567 (7)	0.0212 (2)	
Cl1	0.74487 (8)	0.75170 (12)	0.37010 (7)	0.0249 (2)	
Cl4	0.38501 (9)	0.25191 (13)	0.21270 (9)	0.0336 (3)	
O1	0.7388 (3)	0.6301 (4)	0.5389 (2)	0.0244 (7)	
H1	0.740 (5)	0.705 (7)	0.507 (4)	0.037*	
N1	0.8111 (3)	0.3926 (4)	0.3786 (2)	0.0182 (7)	
N3	0.6939 (2)	0.5043 (5)	0.4853 (2)	0.0183 (6)	
N2	0.6216 (3)	0.2811 (4)	0.3745 (2)	0.0183 (7)	
H2	0.563 (4)	0.289 (6)	0.339 (3)	0.022*	
O2	0.1908 (3)	0.4533 (5)	0.1497 (2)	0.0339 (9)	
H2A	0.226 (6)	0.389 (9)	0.176 (5)	0.051*	
C8	0.6846 (3)	0.3877 (5)	0.5258 (3)	0.0203 (8)	
C5	0.8008 (3)	0.2431 (5)	0.3699 (3)	0.0202 (8)	
N6	0.2619 (3)	0.5471 (4)	0.1318 (2)	0.0212 (8)	
N5	0.4081 (3)	0.6943 (5)	0.0936 (3)	0.0394 (12)	
H5	0.468 (5)	0.736 (8)	0.140 (4)	0.047*	
C12	0.7001 (3)	0.1123 (6)	0.5107 (3)	0.0250 (9)	
H12A	0.660298	0.022653	0.478112	0.030*	
H12B	0.770464	0.117488	0.504159	0.030*	
C14	0.9033 (3)	0.2356 (6)	0.6758 (3)	0.0257 (9)	
C3	0.9925 (4)	0.2136 (6)	0.4131 (3)	0.0301 (11)	
H3	1.054899	0.151943	0.425362	0.036*	
C9	0.7192 (4)	0.3765 (6)	0.6253 (3)	0.0279 (10)	
H9A	0.653362	0.378843	0.638523	0.034*	
H9B	0.764996	0.464264	0.654444	0.034*	
C1	0.9109 (3)	0.4531 (5)	0.4044 (3)	0.0220 (9)	
H1A	0.918464	0.558677	0.410777	0.026*	
C30	0.0369 (4)	0.6416 (7)	−0.1114 (4)	0.0357 (12)	
N4	0.3669 (3)	0.4143 (6)	0.0282 (3)	0.0361 (11)	
C2	1.0041 (3)	0.3660 (6)	0.4222 (3)	0.0277 (10)	
H2B	1.074096	0.411370	0.440161	0.033*	
C25	0.1029 (4)	0.7072 (7)	0.0546 (4)	0.0384 (13)	
H25A	0.095090	0.786671	0.093629	0.046*	
H25B	0.059972	0.619493	0.058673	0.046*	
C7	0.6314 (3)	0.2540 (5)	0.4678 (3)	0.0206 (8)	
C4	0.8897 (4)	0.1499 (6)	0.3860 (3)	0.0278 (10)	
H4	0.880183	0.044699	0.378533	0.033*	
C24	0.2208 (4)	0.6641 (5)	0.0869 (3)	0.0258 (10)	
C6	0.6855 (3)	0.1848 (5)	0.3402 (3)	0.0218 (8)	
H6A	0.649955	0.182503	0.274260	0.026*	
H6B	0.687313	0.081400	0.362311	0.026*	
C10	0.7844 (4)	0.2319 (6)	0.6644 (3)	0.0281 (10)	
H10	0.785669	0.219265	0.725250	0.034*	
C23	0.2999 (4)	0.7724 (5)	0.0732 (4)	0.0365 (12)	
C15	0.9419 (4)	0.3220 (6)	0.6296 (3)	0.0301 (10)	
H15A	1.016603	0.314817	0.638711	0.036*	
H15B	0.894916	0.391186	0.587469	0.036*	
C31	0.0663 (3)	0.5006 (7)	−0.0931 (3)	0.0322 (10)	
H31A	0.053020	0.430999	−0.140077	0.039*	
H31B	0.100801	0.468485	−0.032826	0.039*	
C13	0.5139 (3)	0.2394 (7)	0.4628 (3)	0.0314 (11)	
H13A	0.471161	0.327732	0.433110	0.047*	
H13B	0.516636	0.231714	0.523147	0.047*	
H13C	0.479378	0.149469	0.428806	0.047*	
C11	0.7236 (4)	0.0951 (6)	0.6094 (3)	0.0306 (11)	
H11A	0.768517	0.004325	0.633040	0.037*	
H11B	0.653697	0.081821	0.615749	0.037*	
C16	0.9776 (4)	0.1259 (6)	0.7427 (3)	0.0313 (11)	
H16A	0.980940	0.149758	0.802043	0.047*	
H16B	1.051172	0.132186	0.743621	0.047*	
H16C	0.948965	0.024270	0.726130	0.047*	
C18	0.2741 (6)	0.2441 (10)	−0.0937 (4)	0.0582 (18)	
H18	0.234602	0.153747	−0.115022	0.070*	
C17	0.3151 (5)	0.2832 (8)	−0.0055 (4)	0.0493 (16)	
H17	0.306660	0.213712	0.035033	0.059*	
C26	0.0565 (4)	0.7638 (7)	−0.0421 (4)	0.0423 (14)	
H26	−0.016160	0.810447	−0.054167	0.051*	
C21	0.3804 (4)	0.5139 (10)	−0.0254 (4)	0.0504 (17)	
C28	0.2479 (5)	0.8323 (7)	−0.0237 (4)	0.0486 (16)	
H28A	0.293285	0.915184	−0.030027	0.058*	
H28B	0.247256	0.751483	−0.065083	0.058*	
C32	−0.0176 (7)	0.6926 (9)	−0.2067 (4)	0.068 (2)	
H32A	0.032890	0.756035	−0.220891	0.101*	
H32B	−0.083660	0.749858	−0.215141	0.101*	
H32C	−0.037751	0.605256	−0.246168	0.101*	
C22	0.4373 (5)	0.6511 (10)	0.0162 (4)	0.064 (2)	
H22A	0.416191	0.732765	−0.028357	0.077*	
H22B	0.517365	0.635850	0.038173	0.077*	
C27	0.1312 (6)	0.8883 (7)	−0.0493 (4)	0.0552 (18)	
H27A	0.101814	0.926492	−0.111198	0.066*	
H27B	0.132010	0.972060	−0.009604	0.066*	
C29	0.3272 (7)	0.8968 (8)	0.1403 (5)	0.068 (2)	
H29A	0.368891	0.856492	0.200143	0.102*	
H29B	0.259425	0.942465	0.137844	0.102*	
H29C	0.371164	0.972534	0.126705	0.102*	
C19	0.2927 (7)	0.3391 (10)	−0.1474 (5)	0.069 (2)	
H19	0.270015	0.312455	−0.208304	0.082*	
C20	0.3427 (6)	0.4734 (12)	−0.1196 (4)	0.070 (2)	
H20	0.353114	0.539853	−0.160608	0.084*	

### Atomic displacement parameters (Å^2^)

**Table:**

	*U*^11^	*U*^22^	*U*^33^	*U*^12^	*U*^13^	*U*^23^
Ni1	0.01393 (19)	0.0171 (2)	0.0136 (2)	0.0000 (2)	0.00431 (17)	0.0017 (2)
Ni2	0.0158 (2)	0.0210 (3)	0.0153 (2)	−0.0019 (2)	0.00406 (17)	−0.0011 (2)
Cl2	0.0180 (4)	0.0431 (7)	0.0155 (4)	0.0016 (4)	0.0057 (3)	0.0037 (4)
Cl3	0.0178 (4)	0.0225 (5)	0.0194 (5)	0.0017 (4)	0.0034 (4)	−0.0031 (4)
Cl1	0.0240 (5)	0.0198 (5)	0.0244 (5)	−0.0048 (4)	0.0031 (4)	0.0052 (4)
Cl4	0.0301 (5)	0.0180 (5)	0.0367 (7)	−0.0042 (4)	−0.0027 (5)	0.0021 (5)
O1	0.0275 (15)	0.0233 (17)	0.0205 (16)	0.0014 (13)	0.0077 (13)	−0.0051 (13)
N1	0.0159 (14)	0.0249 (19)	0.0133 (16)	0.0017 (13)	0.0054 (13)	0.0017 (14)
N3	0.0163 (13)	0.0206 (17)	0.0170 (15)	0.0001 (15)	0.0056 (12)	0.0000 (16)
N2	0.0122 (13)	0.0204 (19)	0.0200 (18)	0.0019 (13)	0.0044 (13)	0.0019 (14)
O2	0.0186 (14)	0.052 (3)	0.0312 (19)	−0.0003 (14)	0.0099 (13)	0.0128 (17)
C8	0.0184 (18)	0.026 (2)	0.020 (2)	0.0079 (16)	0.0113 (16)	0.0058 (17)
C5	0.0221 (18)	0.025 (2)	0.0146 (19)	0.0034 (17)	0.0086 (15)	0.0006 (17)
N6	0.0182 (15)	0.028 (2)	0.0163 (17)	0.0011 (13)	0.0055 (14)	−0.0007 (14)
N5	0.029 (2)	0.040 (3)	0.033 (2)	−0.0178 (19)	−0.0040 (18)	0.020 (2)
C12	0.0200 (18)	0.025 (2)	0.027 (2)	−0.0009 (17)	0.0064 (17)	0.0088 (19)
C14	0.0249 (19)	0.033 (3)	0.016 (2)	0.0063 (18)	0.0045 (16)	0.0044 (19)
C3	0.025 (2)	0.036 (3)	0.029 (3)	0.0125 (19)	0.0111 (19)	0.004 (2)
C9	0.032 (2)	0.037 (3)	0.018 (2)	0.011 (2)	0.0145 (18)	0.006 (2)
C1	0.0194 (17)	0.028 (2)	0.019 (2)	0.0006 (16)	0.0077 (15)	0.0024 (17)
C30	0.035 (2)	0.041 (3)	0.033 (3)	0.006 (2)	0.015 (2)	0.000 (2)
N4	0.0223 (18)	0.061 (3)	0.021 (2)	0.0157 (19)	0.0044 (16)	−0.005 (2)
C2	0.0149 (17)	0.041 (3)	0.028 (2)	0.0019 (18)	0.0097 (17)	0.007 (2)
C25	0.033 (2)	0.047 (3)	0.031 (3)	0.022 (2)	0.009 (2)	−0.001 (2)
C7	0.0174 (16)	0.025 (2)	0.022 (2)	0.0014 (16)	0.0105 (15)	0.0077 (18)
C4	0.025 (2)	0.032 (3)	0.027 (2)	0.0106 (18)	0.0114 (18)	0.002 (2)
C24	0.029 (2)	0.025 (2)	0.017 (2)	0.0080 (18)	0.0041 (18)	−0.0052 (18)
C6	0.0243 (19)	0.017 (2)	0.024 (2)	0.0046 (16)	0.0094 (17)	−0.0026 (17)
C10	0.030 (2)	0.034 (3)	0.024 (2)	0.007 (2)	0.0142 (18)	0.012 (2)
C23	0.048 (3)	0.015 (2)	0.032 (3)	−0.003 (2)	0.001 (2)	0.006 (2)
C15	0.0204 (19)	0.036 (3)	0.028 (2)	0.0014 (18)	0.0032 (18)	0.008 (2)
C31	0.0267 (19)	0.033 (2)	0.032 (2)	−0.005 (2)	0.0058 (18)	0.001 (3)
C13	0.0198 (19)	0.040 (3)	0.034 (3)	−0.001 (2)	0.0108 (18)	0.010 (2)
C11	0.026 (2)	0.033 (3)	0.035 (3)	0.0008 (19)	0.015 (2)	0.018 (2)
C16	0.033 (2)	0.037 (3)	0.024 (2)	0.009 (2)	0.0120 (19)	0.011 (2)
C18	0.059 (4)	0.067 (4)	0.032 (3)	0.026 (4)	0.003 (3)	−0.011 (3)
C17	0.040 (3)	0.057 (4)	0.032 (3)	0.025 (3)	−0.004 (2)	−0.020 (3)
C26	0.043 (3)	0.047 (4)	0.028 (3)	0.027 (3)	0.005 (2)	0.002 (2)
C21	0.030 (2)	0.091 (5)	0.031 (3)	0.024 (3)	0.013 (2)	0.016 (3)
C28	0.053 (3)	0.029 (3)	0.043 (3)	−0.005 (3)	−0.002 (3)	0.018 (3)
C32	0.115 (7)	0.050 (4)	0.026 (3)	0.028 (4)	0.016 (4)	0.002 (3)
C22	0.033 (3)	0.113 (7)	0.049 (4)	0.010 (3)	0.020 (3)	0.057 (4)
C27	0.069 (4)	0.029 (3)	0.041 (3)	0.017 (3)	−0.004 (3)	0.007 (3)
C29	0.091 (5)	0.027 (3)	0.051 (4)	0.000 (3)	−0.007 (4)	−0.001 (3)
C19	0.093 (6)	0.071 (5)	0.055 (4)	0.014 (4)	0.044 (4)	−0.020 (4)
C20	0.068 (4)	0.104 (6)	0.040 (3)	0.030 (4)	0.025 (3)	0.035 (4)

### Geometric parameters (Å, º)

**Table:**

Ni1—Cl2	2.4762 (11)	C2—H2B	0.9500
Ni1—Cl3	2.3964 (10)	C25—H25A	0.9900
Ni1—Cl1	2.4408 (12)	C25—H25B	0.9900
Ni1—N1	2.077 (3)	C25—C24	1.495 (6)
Ni1—N3	2.041 (3)	C25—C26	1.536 (8)
Ni1—N2	2.126 (4)	C7—C13	1.545 (5)
Ni2—Cl2	2.4216 (10)	C4—H4	0.9500
Ni2—Cl3	2.4128 (12)	C24—C23	1.516 (7)
Ni2—Cl4	2.4077 (14)	C6—H6A	0.9900
Ni2—N6	2.027 (3)	C6—H6B	0.9900
Ni2—N5	2.103 (4)	C10—H10	1.0000
Ni2—N4	2.095 (4)	C10—C11	1.540 (8)
O1—H1	0.85 (7)	C23—C28	1.550 (8)
O1—N3	1.403 (5)	C23—C29	1.502 (9)
N1—C5	1.345 (6)	C15—H15A	0.9500
N1—C1	1.338 (5)	C15—H15B	0.9500
N3—C8	1.269 (6)	C31—H31A	0.9500
N2—H2	0.77 (5)	C31—H31B	0.9500
N2—C7	1.503 (5)	C13—H13A	0.9800
N2—C6	1.477 (5)	C13—H13B	0.9800
O2—H2A	0.76 (8)	C13—H13C	0.9800
O2—N6	1.385 (5)	C11—H11A	0.9900
C8—C9	1.509 (6)	C11—H11B	0.9900
C8—C7	1.513 (7)	C16—H16A	0.9800
C5—C4	1.385 (6)	C16—H16B	0.9800
C5—C6	1.508 (6)	C16—H16C	0.9800
N6—C24	1.269 (6)	C18—H18	0.9500
N5—H5	0.93 (7)	C18—C17	1.370 (8)
N5—C23	1.517 (7)	C18—C19	1.318 (12)
N5—C22	1.524 (9)	C17—H17	0.9500
C12—H12A	0.9900	C26—H26	1.0000
C12—H12B	0.9900	C26—C27	1.532 (10)
C12—C7	1.555 (6)	C21—C22	1.457 (12)
C12—C11	1.528 (7)	C21—C20	1.465 (10)
C14—C10	1.523 (6)	C28—H28A	0.9900
C14—C15	1.326 (7)	C28—H28B	0.9900
C14—C16	1.506 (7)	C28—C27	1.525 (9)
C3—H3	0.9500	C32—H32A	0.9800
C3—C2	1.373 (8)	C32—H32B	0.9800
C3—C4	1.384 (7)	C32—H32C	0.9800
C9—H9A	0.9900	C22—H22A	0.9900
C9—H9B	0.9900	C22—H22B	0.9900
C9—C10	1.545 (7)	C27—H27A	0.9900
C1—H1A	0.9500	C27—H27B	0.9900
C1—C2	1.396 (6)	C29—H29A	0.9800
C30—C31	1.318 (8)	C29—H29B	0.9800
C30—C26	1.521 (8)	C29—H29C	0.9800
C30—C32	1.503 (8)	C19—H19	0.9500
N4—C17	1.359 (8)	C19—C20	1.360 (13)
N4—C21	1.314 (8)	C20—H20	0.9500
			
Cl3—Ni1—Cl2	84.13 (4)	C8—C7—C13	108.0 (4)
Cl3—Ni1—Cl1	91.99 (4)	C13—C7—C12	110.9 (4)
Cl1—Ni1—Cl2	100.61 (4)	C5—C4—H4	120.7
N1—Ni1—Cl2	88.69 (10)	C3—C4—C5	118.5 (5)
N1—Ni1—Cl3	171.31 (10)	C3—C4—H4	120.7
N1—Ni1—Cl1	94.14 (11)	N6—C24—C25	124.3 (5)
N1—Ni1—N2	79.91 (13)	N6—C24—C23	116.7 (4)
N3—Ni1—Cl2	170.10 (12)	C25—C24—C23	118.8 (4)
N3—Ni1—Cl3	94.30 (9)	N2—C6—C5	110.5 (4)
N3—Ni1—Cl1	89.21 (12)	N2—C6—H6A	109.6
N3—Ni1—N1	91.94 (13)	N2—C6—H6B	109.6
N3—Ni1—N2	77.38 (15)	C5—C6—H6A	109.6
N2—Ni1—Cl2	93.02 (10)	C5—C6—H6B	109.6
N2—Ni1—Cl3	95.56 (9)	H6A—C6—H6B	108.1
N2—Ni1—Cl1	165.04 (11)	C14—C10—C9	114.7 (4)
Cl3—Ni2—Cl2	84.97 (4)	C14—C10—H10	106.7
Cl4—Ni2—Cl2	99.38 (4)	C14—C10—C11	111.4 (4)
Cl4—Ni2—Cl3	93.14 (5)	C9—C10—H10	106.7
N6—Ni2—Cl2	171.72 (12)	C11—C10—C9	110.3 (4)
N6—Ni2—Cl3	92.13 (11)	C11—C10—H10	106.7
N6—Ni2—Cl4	88.51 (11)	N5—C23—C28	112.0 (5)
N6—Ni2—N5	79.29 (16)	C24—C23—N5	109.5 (4)
N6—Ni2—N4	88.11 (15)	C24—C23—C28	108.9 (4)
N5—Ni2—Cl2	93.15 (13)	C29—C23—N5	104.7 (5)
N5—Ni2—Cl3	94.06 (15)	C29—C23—C24	109.9 (5)
N5—Ni2—Cl4	166.04 (12)	C29—C23—C28	111.8 (5)
N4—Ni2—Cl2	93.92 (11)	C14—C15—H15A	120.0
N4—Ni2—Cl3	173.62 (15)	C14—C15—H15B	120.0
N4—Ni2—Cl4	93.24 (16)	H15A—C15—H15B	120.0
N4—Ni2—N5	79.7 (2)	C30—C31—H31A	120.0
Ni2—Cl2—Ni1	91.88 (4)	C30—C31—H31B	120.0
Ni1—Cl3—Ni2	94.09 (4)	H31A—C31—H31B	120.0
N3—O1—H1	111 (4)	C7—C13—H13A	109.5
C5—N1—Ni1	113.6 (3)	C7—C13—H13B	109.5
C1—N1—Ni1	127.6 (3)	C7—C13—H13C	109.5
C1—N1—C5	118.8 (4)	H13A—C13—H13B	109.5
O1—N3—Ni1	121.4 (3)	H13A—C13—H13C	109.5
C8—N3—Ni1	122.2 (3)	H13B—C13—H13C	109.5
C8—N3—O1	115.9 (3)	C12—C11—C10	112.0 (4)
Ni1—N2—H2	93 (4)	C12—C11—H11A	109.2
C7—N2—Ni1	113.6 (3)	C12—C11—H11B	109.2
C7—N2—H2	116 (4)	C10—C11—H11A	109.2
C6—N2—Ni1	104.0 (2)	C10—C11—H11B	109.2
C6—N2—H2	109 (4)	H11A—C11—H11B	107.9
C6—N2—C7	118.1 (3)	C14—C16—H16A	109.5
N6—O2—H2A	105 (5)	C14—C16—H16B	109.5
N3—C8—C9	124.7 (4)	C14—C16—H16C	109.5
N3—C8—C7	116.1 (4)	H16A—C16—H16B	109.5
C9—C8—C7	119.2 (4)	H16A—C16—H16C	109.5
N1—C5—C4	122.3 (4)	H16B—C16—H16C	109.5
N1—C5—C6	115.1 (4)	C17—C18—H18	121.9
C4—C5—C6	122.6 (4)	C19—C18—H18	121.9
O2—N6—Ni2	122.4 (3)	C19—C18—C17	116.1 (8)
C24—N6—Ni2	120.3 (3)	N4—C17—C18	124.7 (7)
C24—N6—O2	116.7 (4)	N4—C17—H17	117.7
Ni2—N5—H5	94 (4)	C18—C17—H17	117.7
C23—N5—Ni2	112.0 (3)	C30—C26—C25	114.3 (5)
C23—N5—H5	115 (4)	C30—C26—H26	107.1
C23—N5—C22	118.6 (4)	C30—C26—C27	112.5 (5)
C22—N5—Ni2	102.9 (4)	C25—C26—H26	107.1
C22—N5—H5	110 (4)	C27—C26—C25	108.5 (5)
H12A—C12—H12B	107.8	C27—C26—H26	107.1
C7—C12—H12A	109.0	N4—C21—C22	116.4 (5)
C7—C12—H12B	109.0	N4—C21—C20	117.2 (8)
C11—C12—H12A	109.0	C22—C21—C20	126.3 (7)
C11—C12—H12B	109.0	C23—C28—H28A	109.2
C11—C12—C7	113.0 (4)	C23—C28—H28B	109.2
C15—C14—C10	124.9 (4)	H28A—C28—H28B	107.9
C15—C14—C16	120.2 (4)	C27—C28—C23	112.2 (6)
C16—C14—C10	114.9 (4)	C27—C28—H28A	109.2
C2—C3—H3	120.1	C27—C28—H28B	109.2
C2—C3—C4	119.7 (4)	C30—C32—H32A	109.5
C4—C3—H3	120.1	C30—C32—H32B	109.5
C8—C9—H9A	109.2	C30—C32—H32C	109.5
C8—C9—H9B	109.2	H32A—C32—H32B	109.5
C8—C9—C10	112.2 (4)	H32A—C32—H32C	109.5
H9A—C9—H9B	107.9	H32B—C32—H32C	109.5
C10—C9—H9A	109.2	N5—C22—H22A	109.7
C10—C9—H9B	109.2	N5—C22—H22B	109.7
N1—C1—H1A	119.0	C21—C22—N5	110.0 (4)
N1—C1—C2	122.1 (4)	C21—C22—H22A	109.7
C2—C1—H1A	119.0	C21—C22—H22B	109.7
C31—C30—C26	124.8 (5)	H22A—C22—H22B	108.2
C31—C30—C32	120.1 (5)	C26—C27—H27A	109.3
C32—C30—C26	115.1 (5)	C26—C27—H27B	109.3
C17—N4—Ni2	126.7 (4)	C28—C27—C26	111.5 (5)
C21—N4—Ni2	113.2 (4)	C28—C27—H27A	109.3
C21—N4—C17	119.9 (5)	C28—C27—H27B	109.3
C3—C2—C1	118.6 (4)	H27A—C27—H27B	108.0
C3—C2—H2B	120.7	C23—C29—H29A	109.5
C1—C2—H2B	120.7	C23—C29—H29B	109.5
H25A—C25—H25B	107.9	C23—C29—H29C	109.5
C24—C25—H25A	109.2	H29A—C29—H29B	109.5
C24—C25—H25B	109.2	H29A—C29—H29C	109.5
C24—C25—C26	112.1 (4)	H29B—C29—H29C	109.5
C26—C25—H25A	109.2	C18—C19—H19	118.5
C26—C25—H25B	109.2	C18—C19—C20	123.0 (7)
N2—C7—C8	109.8 (3)	C20—C19—H19	118.5
N2—C7—C12	112.5 (3)	C21—C20—H20	120.5
N2—C7—C13	107.1 (3)	C19—C20—C21	119.0 (7)
C8—C7—C12	108.5 (3)	C19—C20—H20	120.5
			
Ni1—N1—C5—C4	−178.5 (3)	N4—C21—C20—C19	2.4 (9)
Ni1—N1—C5—C6	2.4 (4)	C2—C3—C4—C5	−0.9 (7)
Ni1—N1—C1—C2	177.8 (3)	C25—C24—C23—N5	−168.7 (4)
Ni1—N3—C8—C9	171.5 (3)	C25—C24—C23—C28	−45.9 (6)
Ni1—N3—C8—C7	−10.6 (5)	C25—C24—C23—C29	76.9 (6)
Ni1—N2—C7—C8	−7.7 (4)	C25—C26—C27—C28	59.6 (6)
Ni1—N2—C7—C12	−128.6 (3)	C7—N2—C6—C5	−83.5 (4)
Ni1—N2—C7—C13	109.3 (4)	C7—C8—C9—C10	47.9 (5)
Ni1—N2—C6—C5	43.4 (4)	C7—C12—C11—C10	−57.9 (5)
Ni2—N6—C24—C25	173.2 (4)	C4—C5—C6—N2	148.6 (4)
Ni2—N6—C24—C23	−12.5 (6)	C4—C3—C2—C1	0.6 (7)
Ni2—N5—C23—C24	−13.2 (5)	C24—C25—C26—C30	73.6 (6)
Ni2—N5—C23—C28	−134.2 (4)	C24—C25—C26—C27	−52.8 (6)
Ni2—N5—C23—C29	104.5 (5)	C24—C23—C28—C27	49.5 (6)
Ni2—N5—C22—C21	44.6 (5)	C6—N2—C7—C8	114.5 (4)
Ni2—N4—C17—C18	174.2 (4)	C6—N2—C7—C12	−6.5 (5)
Ni2—N4—C21—C22	5.4 (6)	C6—N2—C7—C13	−128.6 (4)
Ni2—N4—C21—C20	−178.0 (4)	C6—C5—C4—C3	180.0 (4)
O1—N3—C8—C9	−0.7 (6)	C23—N5—C22—C21	−79.6 (6)
O1—N3—C8—C7	177.3 (3)	C23—C28—C27—C26	−59.7 (7)
N1—C5—C4—C3	1.0 (7)	C15—C14—C10—C9	−24.5 (7)
N1—C5—C6—N2	−32.3 (5)	C15—C14—C10—C11	101.6 (6)
N1—C1—C2—C3	−0.2 (7)	C31—C30—C26—C25	−6.7 (8)
N3—C8—C9—C10	−134.2 (4)	C31—C30—C26—C27	117.5 (6)
N3—C8—C7—N2	11.5 (5)	C11—C12—C7—N2	172.3 (3)
N3—C8—C7—C12	134.8 (4)	C11—C12—C7—C8	50.6 (4)
N3—C8—C7—C13	−104.9 (4)	C11—C12—C7—C13	−67.8 (5)
O2—N6—C24—C25	1.3 (6)	C16—C14—C10—C9	158.2 (4)
O2—N6—C24—C23	175.6 (4)	C16—C14—C10—C11	−75.7 (5)
C8—C9—C10—C14	77.6 (5)	C18—C19—C20—C21	1.5 (12)
C8—C9—C10—C11	−49.1 (5)	C17—N4—C21—C22	−179.7 (5)
C5—N1—C1—C2	0.3 (6)	C17—N4—C21—C20	−3.0 (7)
N6—C24—C23—N5	16.7 (6)	C17—C18—C19—C20	−4.4 (11)
N6—C24—C23—C28	139.5 (5)	C26—C25—C24—N6	−136.7 (5)
N6—C24—C23—C29	−97.7 (5)	C26—C25—C24—C23	49.2 (7)
N5—C23—C28—C27	170.8 (5)	C21—N4—C17—C18	0.0 (8)
C14—C10—C11—C12	−72.9 (5)	C32—C30—C26—C25	174.8 (6)
C9—C8—C7—N2	−170.4 (3)	C32—C30—C26—C27	−60.9 (7)
C9—C8—C7—C12	−47.1 (5)	C22—N5—C23—C24	106.3 (6)
C9—C8—C7—C13	73.2 (5)	C22—N5—C23—C28	−14.6 (6)
C9—C10—C11—C12	55.6 (5)	C22—N5—C23—C29	−135.9 (6)
C1—N1—C5—C4	−0.6 (6)	C22—C21—C20—C19	178.6 (6)
C1—N1—C5—C6	−179.7 (4)	C29—C23—C28—C27	−72.1 (7)
C30—C26—C27—C28	−67.9 (6)	C19—C18—C17—N4	3.8 (9)
N4—C21—C22—N5	−35.0 (7)	C20—C21—C22—N5	148.7 (6)

### Hydrogen-bond geometry (Å, º)

**Table:**

*D*—H···*A*	*D*—H	H···*A*	*D*···*A*	*D*—H···*A*
O1—H1···Cl1	0.85 (7)	2.32 (6)	3.009 (4)	139 (6)
N2—H2···Cl4	0.77 (5)	2.46 (5)	3.209 (4)	166 (5)
O2—H2*A*···Cl4	0.76 (8)	2.31 (7)	2.978 (4)	147 (7)
C3—H3···O1^i^	0.95	2.58	3.432 (5)	149
C1—H1*A*···Cl1	0.95	2.75	3.369 (5)	124
C6—H6*A*···Cl2	0.99	2.76	3.309 (5)	115
C11—H11*B*···Cl3^ii^	0.99	2.64	3.573 (5)	156
C17—H17···Cl4	0.95	2.69	3.327 (6)	125
C26—H26···O2^iii^	1.00	2.56	3.489 (6)	154
C22—H22*B*···Cl2	0.99	2.81	3.352 (6)	115
C19—H19···Cl1^iv^	0.95	2.64	3.570 (7)	167

Symmetry codes: (i) −*x*+2, *y*−1/2, −*z*+1; (ii) −*x*+1, *y*−1/2, −*z*+1; (iii) −*x*, *y*+1/2, −*z*; (iv) −*x*+1, *y*−1/2, −*z*.

## References

[bb1] Anastas, P. T. & Zimmerman, J. B. (2013). *Environ. Sci. Technol.* **37**, 95*A*–101*A*. 10.1021/es032373g 12666905

[bb2] Benabdelouahab, Y., Muñoz-Moreno, L., Frik, M., de la Cueva-Alique, I., El Amrani, M. A., Contel, M., Bajo, A. M., Cuenca, T. & Royo, E. (2015). *Eur. J. Inorg. Chem.* pp. 2295–2307. 10.1002/ejic.201500097 PMC4862618 27175101

[bb3] Bruker (2019). *APEX2* and *SAINT*. Bruker AXS Inc., Madison Wisconsin, USA.

[bb4] Chahboun, G., Brito, J. A., Royo, B., El Amrani, M. A., Gómez-Bengoa, E., Mosquera, M. E. G., Cuenca, T. & Royo, E. (2012). *Eur. J. Inorg. Chem.* pp. 2940–2949.

[bb5] Cheng, T.-P., Liao, B.-S., Liu, Y.-H., Peng, S.-M. & Liu, S.-T. (2012). *Dalton Trans.* **41**, 3468–3473. 10.1039/c2dt11398h 22302140

[bb6] Cueva-Alique, I. de la, Muñoz-Moreno, L., de la Torre-Rubio, E., Bajo, A. M., Gude, L., Cuenca, T. & Royo, E. (2019). *Dalton Trans.* **48**, 14279–14293. 10.1039/c9dt02873k 31482936

[bb7] Dolomanov, O. V., Bourhis, L. J., Gildea, R. J., Howard, J. A. K. & Puschmann, H. (2009). *J. Appl. Cryst.* **42**, 339–341.

[bb8] Elalami, M. S., Dahdouh, A. A., Mansour, A. I., ElAmrani, M. A., Suisse, I., Mortreux, A. & Agbossou-Niedercorn, F. (2009). *C. R. Chim.* **12**, 1253–1258.

[bb9] El Alami, M. S. I., El Amrani, M. A., Agbossou-Niedercorn, F., Suisse, I. & Mortreux, A. (2015). *Chem. Eur. J.* **21**, 1398–1413. 10.1002/chem.201404303 25359663

[bb10] Gawley, R. E. & Aubé, J. (2012). *Principles and applications of asymmetric synthesis*, 2nd ed. Amsterdam: Elsevier Science

[bb11] El Alami, M. S. I., El Amrani, M. A., Dahdouh, A., Roussel, P., Suisse, I. & Mortreux, A. (2012). *Chirality*, **24**, 675–682. 10.1002/chir.22073 22711228

[bb12] Krause, L., Herbst-Irmer, R., Sheldrick, G. M. & Stalke, D. (2015). *J. Appl. Cryst.* **48**, 3–10. 10.1107/S1600576714022985 PMC4453166 26089746

[bb13] Ojima, I. (2010). *Catalytic asymmetric synthesis*, 3rd ed. Hoboken: Wiley

[bb14] Parsons, S., Flack, H. D. & Wagner, T. (2013). *Acta Cryst.* B**69**, 249–259. 10.1107/S2052519213010014 PMC3661305 23719469

[bb15] Sheldrick, G. M. (2015*a*). *Acta Cryst.* A**71**, 3–8.

[bb16] Sheldrick, G. M. (2015*b*). *Acta Cryst.* C**71**, 3–8.

[bb17] Zheng, L., Zhang, S., Li, K., Chen, W., Chen, Y., Xu, B., Hu, B., Li, Y. & Li, W. (2010). *J. Mol. Struct.* **984**, 153–156.

